# Independent association of atherogenic dyslipidaemia with all‐cause mortality in individuals with type 2 diabetes and modifying effect of gender: a prospective cohort study

**DOI:** 10.1186/s12933-021-01224-7

**Published:** 2021-01-30

**Authors:** Emanuela Orsi, Giuseppe Penno, Anna Solini, Enzo Bonora, Cecilia Fondelli, Roberto Trevisan, Monica Vedovato, Franco Cavalot, Susanna Morano, Marco G. Baroni, Antonio Nicolucci, Giuseppe Pugliese, for the Renal Insufficiency And Cardiovascular Events (RIACE) Study Group, Giuseppe Pugliese, Giuseppe Penno, Anna Solini, Enzo Bonora, Emanuela Orsi, Roberto Trevisan, Luigi Laviola, Antonio Nicolucci

**Affiliations:** 1 Diabetes Unit, IRCCS “Cà Granda - Ospedale Maggiore Policlinico” Foundation, Milan, Italy; 2 Department of Clinical and Experimental Medicine, University of Pisa, Pisa, Italy; 3 Department of Surgical, Medical, Molecular and Critical Area Pathology, University of Pisa, Pisa, Italy; 4 Division of Endocrinology, Diabetes and Metabolism, University and Hospital Trust of Verona, Verona, Italy; 5 Diabetes Unit, University of Siena, Siena, Italy; 6 Endocrinology and Diabetes Unit, Azienda Ospedaliera Papa Giovanni XXIII, Bergamo, Italy; 7 Department of Clinical and Experimental Medicine, University of Padua, Padua, Italy; 8 Department of Clinical and Biological Sciences, University of Turin, Orbassano, Italy; 9 Department of Experimental Medicine, “La Sapienza” University, Rome, Italy; 10 Department of Clinical Medicine, Public Health, and Life and Environment Sciences, University of L’Aquila, L’Aquila, Italy; 11 Centre for Outcomes Research and Clinical Epidemiology (CORESEARCH), Pescara, Italy; 12 Department of Clinical and Molecular Medicine, “La Sapienza” University, Via di Grottarossa, 1035-1039, 00189 Rome, Italy

**Keywords:** Type 2 diabetes, All-cause mortality, Atherogenic dyslipidaemia, Triglycerides, HDL cholesterol, Triglyceride:HDL cholesterol ratio

## Abstract

**Background:**

Atherogenic dyslipidaemia has been implicated in the residual risk for cardiovascular morbidity and mortality, which remains despite attainment of LDL cholesterol goals especially in individuals with type 2 diabetes. However, its relationship with all-cause death has not been sufficiently explored. This analysis evaluated the independent association of increased triglycerides and triglyceride:HDL cholesterol ratio (TG:HDL) and decreased HDL cholesterol with total mortality and the possible modifying effect of gender in a large cohort of patients with type 2 diabetes.

**Methods:**

This observational, prospective study enrolled 15,773 patients in 19 Diabetes Clinics throughout Italy in the years 2006–2008. Triglycerides and total and HDL cholesterol were measured by colorimetric enzymatic methods. Vital status was retrieved on 31 October 2015 for 15,656 patients (99.3%). Participants were stratified by quartiles of triglycerides, HDL cholesterol, and TG:HDL.

**Results:**

There were 3,602 deaths over a follow-up 7.42 ± 2.05 years (31.0 × 1000 person-years). In the unadjusted analyses, the highest TG:HDL (but not triglyceride) and the lowest HDL cholesterol quartile were associated with increased death rate and mortality risk. When sequentially adjusting for confounders, including total, LDL, or non-HDL cholesterol and lipid-lowering treatment, mortality risk was significantly higher in the highest triglyceride (hazard ratio 1.167 [95% confidence interval 1.055–1.291], *p* = 0.003) and TG:HDL (1.192 [1.082–1.314], *p* < 0.0001) and the lowest HDL cholesterol (1.232 [1.117–1.360], *p* < 0.0001) quartile, though the association of triglycerides and HDL cholesterol disappeared after further adjustment for each other. Interaction with gender was significant only for HDL cholesterol (*p* = 0.0009). The relationship with death was stronger for triglycerides in males and HDL cholesterol in females, with these associations remaining significant even after adjustment for HDL cholesterol (1.161 [1.019–1.324], *p* = 0.025, for the highest vs the lowest triglyceride quartile) and triglycerides (1.366 [1.176–1.587], *p* < 0.0001, for the lowest vs the highest HDL cholesterol quartile).

**Conclusions:**

In patients with type 2 diabetes, higher triglycerides and TG:HDL and lower HDL cholesterol were independently associated with increased all-cause mortality, with a modifying effect of gender for triglycerides and HDL cholesterol. These data suggest that atherogenic dyslipidaemia, especially TG:HDL, may serve as predictor of all-cause death in these individuals.

*Trial registration* ClinicalTrials.gov, NCT00715481, 15 July, 2008

## Background

Diabetes is associated with excess mortality mainly from cardiovascular disease (CVD) [1]. Increased LDL cholesterol is a major risk factor for atherosclerotic CVD, as consistently shown by observational and Mendelian randomization studies [2] as well as by intervention trials with statins and other cholesterol reducing agents [3], with no difference between patients with and without diabetes [2, 4]. However, despite attainment of LDL cholesterol goals, a residual CVD risk remains. At least part of this risk has been attributed to increased levels of other apolipoprotein (Apo) B-containing lipoproteins, including smaller triglycerides-rich lipoproteins (TRLs) and their remnant particles, and the associated inverse changes in HDL cholesterol [5]. This lipid pattern, called atherogenic dyslipidaemia, is a common feature in conditions characterized by insulin resistance, such as type 2 diabetes and the other abnormalities clustering in the metabolic syndrome [5]. In individuals with atherogenic dyslipidaemia, relative CVD risk is much greater in women [6], a finding that may explain the gender differences in CVD risk observed in diabetic patients [7].

In addition, diabetes is associated with an increased risk of death from other causes [1], especially cancer [8], infections [9], and liver disease [10], which also contribute to the excess total mortality of diabetic individuals. However, the existing literature on the relationship of lipid levels with all-cause death is not as abundant and univocal as that regarding CVD mortality. Previous studies have found increased total mortality at both ends of the lipid spectrum in the general population, with differences depending on the characteristic of the study sample, i.e., general versus disease-specific population, younger versus older individuals, and Caucasian versus other ethnicities, and reflecting differences in causes of death and circulating lipid concentrations. Among Caucasians, individuals with higher total and LDL cholesterol levels had greater mortality risk [11, 12], though a few studies found U-shaped or inverse relationships [13, 14], whereas, in East Asian populations [15, 16] and Pima Indians [17], the highest risk for mortality was reported in people with low total cholesterol. The U-shaped or inverse relationships of lipids (positive for HDL cholesterol) with mortality have been associated with older age [11, 18] and smoking [13] and attributed to the contribution of deaths from cancer, infections, and alcohol-related disorders (including liver disease and accidents) [11–13, 15]. However, low lipid levels appear to be a consequence of these conditions rather than playing a causal role in the associated increase in mortality [19].

In people with type 2 diabetes, intervention trials with statins have shown that reduction of LDL cholesterol levels was associated with decreased mortality from CVD, but not from any cause [20]. In a previous report from the Renal Insufficiency and Cardiovascular Events (RIACE) cohort, we showed that total and LDL cholesterol were inversely associated with all-cause mortality, a finding attributed to an indication effect. In fact, prevalence of statin treatment at baseline was less than 50%, suggesting that it was restricted to high-risk patients, who therefore showed higher mortality rate associated with lower cholesterol levels than those at lower risk [21]. This interpretation is consistent with previous data indicating that the association between cholesterol and CVD in contemporary studies may be attenuated by the preferential use of statins by high-risk individuals [22]. Conversely, the association between the abnormalities of lipid fractions clustering in atherogenic dyslipidaemia and all-cause mortality has not been extensively investigated in individuals with type 2 diabetes and the few studies addressing this issue have provided contrasting findings for HDL cholesterol [23–26] and no evidence of gender differences.

The present analysis aimed at assessing the independent association of the abnormal lipid profile characterizing atherogenic dyslipidaemia (i.e., increased triglycerides, decreased HDL cholesterol, and increased ratio of the two lipid fractions) with death from any cause and the possible modifying effect of gender in the large RIACE cohort of individuals with type 2 diabetes.

## Methods

### Design

The RIACE Italian Multicentre Study is an observational, prospective, cohort study on the impact of estimated glomerular filtration rate (eGFR) on morbidity and mortality in individuals with type 2 diabetes [21, 27].

### Patients

The study population included 15,773 Caucasian patients (after excluding 160 individuals with missing or implausible values), consecutively attending 19 hospital-based, tertiary referral Diabetes Clinics of the National Health Service throughout Italy in the years 2006–2008. Exclusion criteria were dialysis or renal transplantation.

### All-cause mortality

The vital status of study participants on 31 October 2015 was verified by interrogating the Italian Health Card database (http://sistemats1.sanita.finanze.it/wps/portal/), which provides updated and reliable information on all current Italian residents [28].

### Baseline measurements

Baseline data were collected using a standardized protocol across participating centres [21, 27].

Participants underwent a structured interview in order to collect the following information: age at the time of the interview, smoking status, known diabetes duration, co-morbidities, and current glucose-, lipid-, and blood pressure (BP)-lowering treatments.

Body mass index (BMI) was calculated from weight and height, whereas waist circumference was estimated from log-transformed BMI values; BP was measured with a sphygmomanometer with the patients seated with the arm at the heart level.

Haemoglobin A_1c_ (HbA_1c_) was measured by HPLC using DCCT-aligned methods; triglycerides and total and HDL cholesterol were determined in fasting blood samples by colorimetric enzymatic methods. The triglyceride:HDL cholesterol ratio (TG:HDL) was then calculated by dividing triglyceride for HDL cholesterol levels (both in mg/dl) and LDL cholesterol concentration was estimated using the Friedewald formula.

The presence of diabetic kidney disease (DKD) was assessed by measuring albuminuria and serum creatinine, as previously detailed [23, 29]. Albumin excretion rate was obtained from 24-h urine collections or calculated from albumin-to-creatinine ratio in early-morning, first-voided urine samples, using a conversion formula developed in patients with type 1 diabetes and preliminarily validated in a subgroup of RIACE participants. Albuminuria was measured in fresh urine samples by immunonephelometry or immunoturbidimetry, in the absence of interfering clinical conditions. One-to-three measurements for each patient were obtained; in cases of multiple measurements, the geometric mean of 2–3 values was used for analysis. In individuals with multiple measurements, the concordance rate between the first value and the geometric mean was > 90% for all albuminuria categories [29]. Serum (and urine) creatinine was measured by the modified Jaffe method, traceable to IDMS, and eGFR was calculated using the Chronic Kidney Disease Epidemiology Collaboration equation [21]. Patients were then classified into Kidney Disease: Improving Global Outcomes categories of albuminuria (A1 to A3) and eGFR (G1 to G5) and assigned to one of the following DKD phenotypes: no DKD (i.e., A1G1-A1G2), albuminuria alone (albuminuric DKD with preserved eGFR, i.e., A2G1-A2G2-A3G1-A3G2), reduced eGFR alone (non-albuminuric DKD, i.e., A1G3-A1G4-A1G5), or both albuminuria and reduced eGFR (albuminuric DKD with reduced eGFR, i.e., A2G3-A2G4-A2G5-A3G3-A3G4-A3G5), as previously reported [21].

In each centre, the presence of diabetic retinopathy (DR) was assessed by an expert ophthalmologist by dilated fundoscopy [30]. Patients with mild or moderate non-proliferative DR were classified as having non-advanced DR, whereas those with severe non-proliferative DR, proliferative DR, or maculopathy were grouped into the advanced, sight threatening DR category. DR grade was assigned based on the worse eye.

Previous major acute CVD events, including myocardial infarction; stroke; foot ulcer/gangrene/amputation; and coronary, carotid, and lower limb revascularization, were adjudicated based on hospital discharge records by an ad hoc committee in each centre [31].

### Statistical analysis

For the purpose of the current analysis, the whole RIACE cohort and men and women separately were divided into quartiles of triglycerides, HDL cholesterol, and TG:HDL.

Data are expressed as mean ± SD or median (interquartile range) for continuous variables, and number of cases and percentage for categorical variables. Comparisons among quartiles were performed by one-way ANOVA or Kruskal–Wallis test, according to the parametric or non-parametric distribution of continuous variables, followed by Bonferroni correction or Mann–Whitney test, respectively, for post-hoc comparisons, and by Pearson’s χ^2^ test for categorical variables.

Crude mortality rates were described as events per 1000 patient-years, with 95% exact Poisson confidence intervals (CIs) and adjusted for age and gender by a Poisson regression model. Kaplan–Meier survival probabilities for all-cause mortality were estimated according to the above categorizations and differences were analysed using the log-rank statistic. The hazard ratios (HRs) and their 95% CIs were estimated by Cox proportional hazards regression, using the lowest triglyceride, the highest HDL cholesterol or the lowest triglyceride TG:HDL quartile as reference. These analyses were adjusted for age and gender (model 1), plus CVD risk factors, i.e., smoking, diabetes duration, HbA_1c_, BMI, total cholesterol (or, in alternative, LDL or non-HDL cholesterol), and systolic and diastolic BP, and treatments, i.e., anti-hyperglycaemic, lipid-lowering, and anti-hypertensive therapy (model 2), plus presence of complications, i.e., DKD phenotypes, DR grade and any CVD, and/or severe comorbidity(ies), i.e., chronic obstructive pulmonary disease, chronic liver disease and/or cancer (model 3), and, for triglycerides and HDL cholesterol quartiles only, plus HDL cholesterol and triglyceride levels, respectively, as continuous variables (model 4). Covariates were selected a priori, as all of them potentially affect mortality. Appropriate tests were applied for assessing the interaction between gender and quartiles of triglycerides, HDL cholesterol, and TG:HDL and the analyses were then replicated separately for men and women. Finally, all the above analyses were rerun after substituting LDL cholesterol for total cholesterol and the association between HDL cholesterol and mortality was evaluated also among individuals falling in the lowest quartile of LDL cholesterol.

All *p* values were two-sided, and a *p* < 0.05 was considered statistically significant. Statistical analyses were performed using SPSS version 13.0 (SPSS Inc., Chicago, IL, USA).

## Results

Valid information on vital status was retrieved for 15,656 participants (99.3% of the cohort). At the time of the census, 3,602 (23.0%) individuals had died; death rate was 31.0 per 1000 person-years (95% CI 30.0–32.0) over a follow-up of 7.4 ± 2.1 years [21].

### Association of triglycerides, HDL cholesterol and TG:HDL with all-cause mortality in the whole cohort

The clinical features of study participants stratified by quartiles of triglycerides, HDL cholesterol, and TG:HDL are shown in Additional file 1: Tables S1–S3. Patients falling in the fourth (highest) quartile of triglycerides or TG:HDL or in the first (lowest) quartile of HDL cholesterol were younger, less frequently smokers, and had shorter diabetes duration, but worse CVD risk profile and higher prevalence of complications than those falling in the first (lowest) quartile of triglycerides and TG:HDL or in the fourth (highest) quartile of HDL cholesterol.

**Fig. 1:**
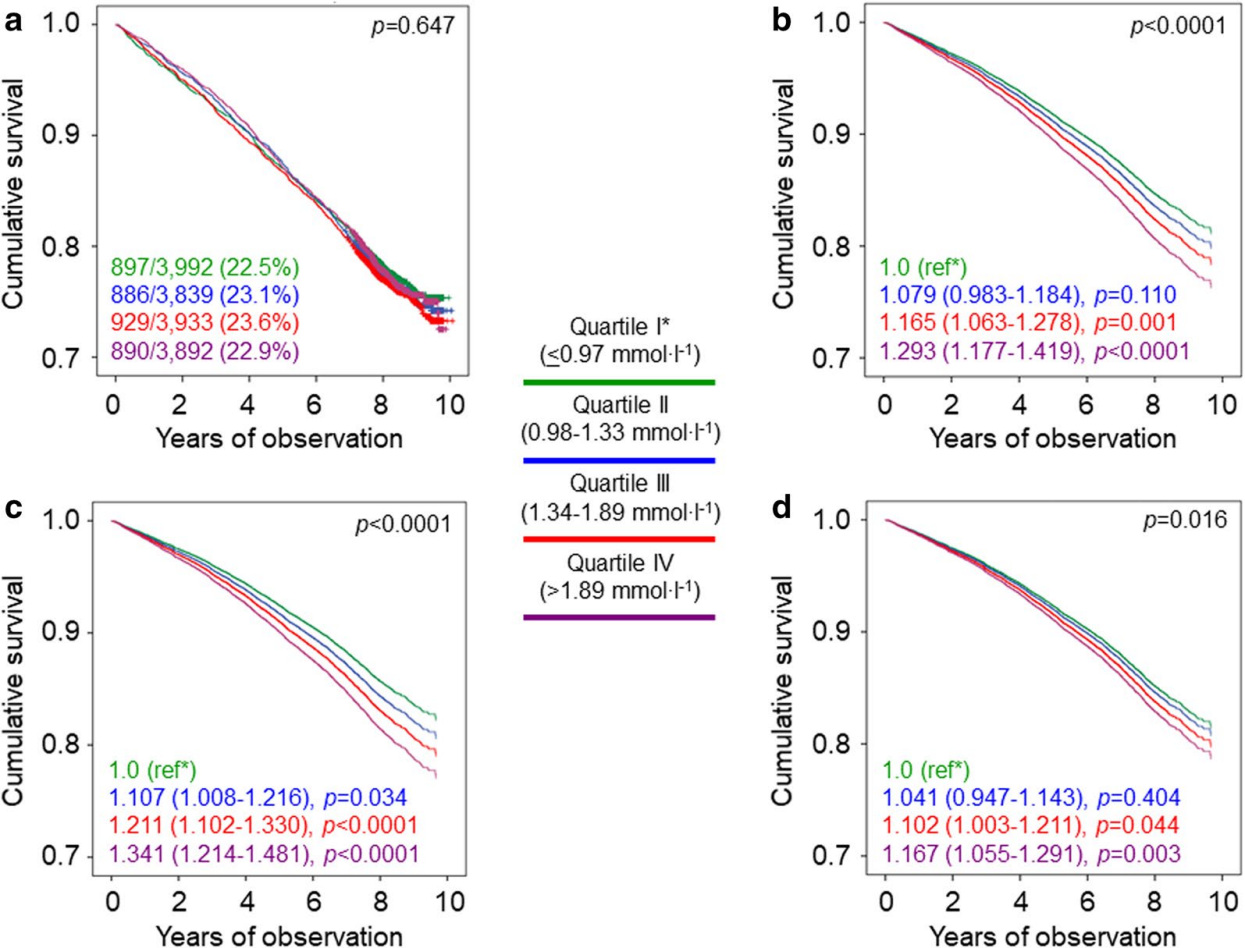
Survival analysis by quartiles of triglycerides in the whole cohort. Cumulative survival by Kaplan Meier-analysis (**a**) and Cox proportional hazards regression, adjusted for age and gender (**b**), plus CVD risk factors (**c**) plus complications/comorbidities (**d**), according to quartiles of triglycerides. Numbers (percentages) of deaths and HRs (95% CI) for mortality are shown for each group. *HR* hazard ratio, *CI* confidence interval

Crude mortality rates were similar among quartiles of triglycerides; however, when adjusted for age and gender, mortality rates became significantly higher in quartiles III and IV versus quartile I (Table 1). Kaplan–Meier estimates (Fig. 1a) and unadjusted HRs (not shown) did not differ among quartiles of triglycerides. When sequentially adjusting for confounders (models 1–3), mortality risk became significantly higher in quartiles III and IV versus quartile I (Fig. 1b–d), but differences disappeared after further adjustment for HDL cholesterol (HR, 1.039 [95% CI 0.940–1.149], *p* = 0.450 for quartile III, and 1.066 [0.952–1.194], *p* = 0.269 for quartile IV).

**Table 1:** Mortality rates in study participants with valid information on vital status on October 31 2015, stratified by quartiles of triglycerides, HDL cholesterol, and TG:HDL

	N	Events	Percent events	Events per 1000 patient-years (95% CI), unadjusted	*p*	Events per 1000 patient-years (95% CI), age- & gender-adjusted	*p*
Triglycerides					0.666		< 0.0001
I	3992	897	22.5	30.25 (28.33–32.29)	Ref	11.02 (9.60–12.64)	Ref
II	3839	886	23.1	31.09 (29.11–33.20)	0.563	11.82 (10.35–13.50)	0.138
III	3933	929	23.6	32.03 (30.03–34.15)	0.222	12.76 (11.20–14.54)	0.002
IV	3892	890	22.9	30.76 (28.80–32.85)	0.722	14.10 (12.39–16.05)	< 0.0001
HDL cholesterol					< 0.0001		< 0.0001
I	4025	1061	26.4	36.53 (34.39–38.79)	< 0.0001	18.16 (15.81–20.86)	< 0.0001
II	3982	881	22.1	29.68 (27.78–31.70)	0.566	13.68 (11.94–15.68)	0.008
III	3769	818	21.7	29.01 (27.09–31.07)	0.921	12.55 (10.98–14.36)	0.392
IV	3880	842	21.7	28.87 (26.98–30.88)	Ref	12.03 (10.60–13.66)	Ref
TG:HDL					0.056		< 0.0001
I	3914	863	22.1	29.48 (27.58–31.51)	Ref	11.02 (9.65–12.59)	Ref
II	3916	869	22.2	29.79 (27.87–31.83)	0.829	11.83 (10.36–13.50)	0.143
III	3915	927	23.7	32.12 (30.12–34.25)	0.070	13.18 (11.56–15.03)	< 0.0001
IV	3911	943	24.1	32.76 (30.73–34.91)	0.025	15.51 (13.60–17.70)	< 0.0001

*TG:HDL* triglyceride:HDL cholesterol ratio, *CI* confidence interval

**Fig. 2:**
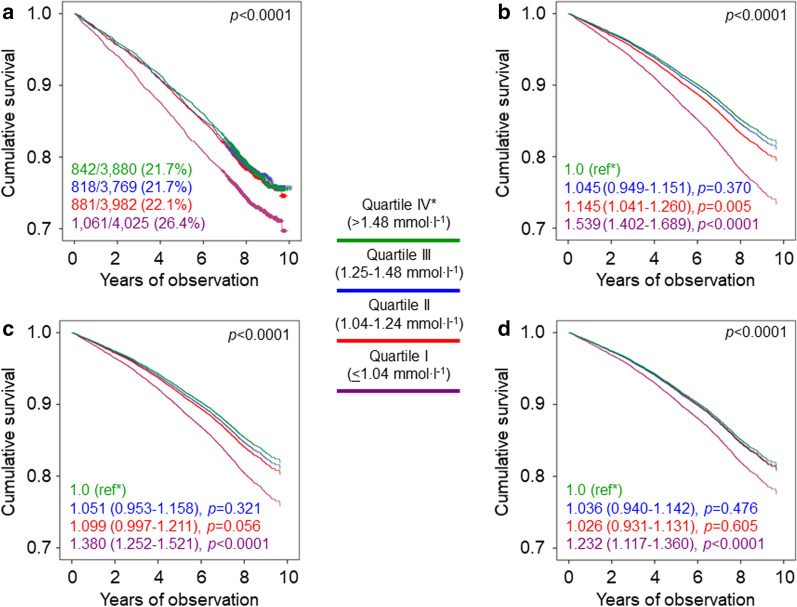
Survival analysis by quartiles of HDL cholesterol in the whole cohort. Survival analysis by quartiles of HDL cholesterol in the whole cohort. Cumulative survival by Kaplan Meier-analysis (**a**) and Cox proportional hazards regression, adjusted for age and gender (**b**), plus CVD risk factors (**c**) plus complications/comorbidities (**d**), according to quartiles of HDL cholesterol. Numbers (percentages) of deaths and HRs (95% CI) for mortality are shown for each group. *HR* hazard ratio, *CI* confidence interval

Crude and age- and gender-adjusted mortality rates were higher for quartile I of HDL cholesterol versus quartile IV, whereas only the adjusted rates were higher for quartile II versus quartile IV (Table 1). Kaplan–Meier estimates (Fig. 2a) and unadjusted (not shown) and adjusted (models 1–3, Fig. 2b–d) HRs were also higher for quartile I versus quartile IV, except after further adjustment for triglycerides (0.968 [0.786–1.191], *p* = 0.756).

**Fig. 3:**
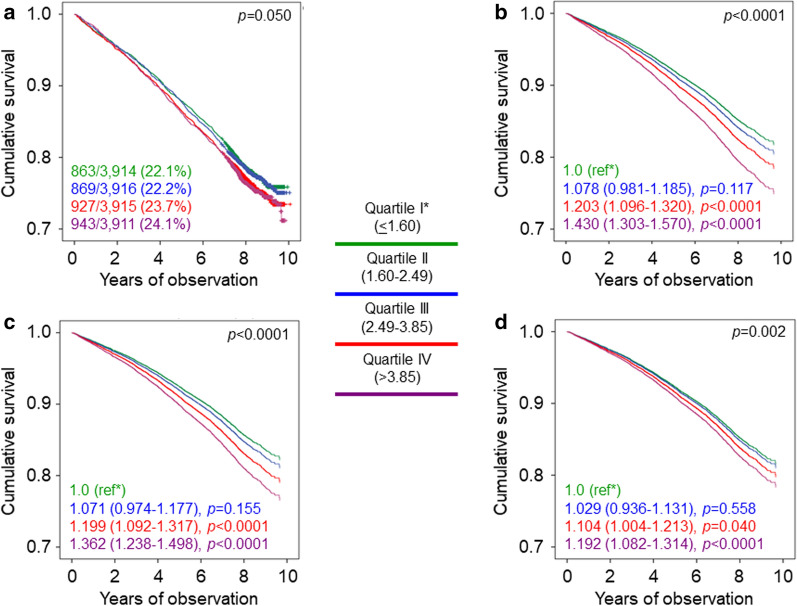
Survival analysis by quartiles of TG:HDL in the whole cohort. Cumulative survival by Kaplan Meier-analysis (**a**) and Cox proportional hazards regression, adjusted for age and gender (**b**), plus CVD risk factors (**c**) plus complications/comorbidities (**d**), according to quartiles of TG:HDL ratio. Numbers (percentages) of deaths and HRs (95% CI) for mortality are shown for each group. *TG:HDL* triglyceride:HDL cholesterol ratio, *HR* hazard ratio, *CI* confidence interval

Crude and age- and gender-adjusted mortality rates were higher for quartile IV versus quartile I of TG:HDL, whereas only the adjusted rates were higher for quartile III versus quartile I (Table 1). Kaplan–Meier estimates (Fig. 3a) and unadjusted (not shown) and adjusted (Fig. 3b–d) HRs were also higher for quartile IV versus quartile I, whereas only the adjusted HRs were higher for quartile III versus quartile I.

Results did not change when either LDL or non-HDL cholesterol was substituted for total cholesterol (not shown). Moreover, the inverse association of HDL cholesterol with all-cause mortality was detected also in the lowest quartile of LDL cholesterol (≤ 2.21 mmol/L) cholesterol (not shown).

### Association of triglycerides, HDL cholesterol and TG:HDL with all-cause mortality by gender

Interaction with gender was significant for HDL cholesterol (*p* = 0.009), but not for triglyceride and TG:HDL quartiles. The clinical features of male and female participants stratified by quartiles of triglycerides, HDL cholesterol, and TG:HDL did not differ appreciably compared with those of the whole cohort (not shown).

No differences among quartiles of triglycerides were observed in crude mortality rates in both males and females, whereas age-adjusted death rates were increased for quartiles III and IV versus quartile I only in male participants (Table 2).

**Table 2:** Mortality rates in male and female study participants with valid information on vital status on October 31 2015, stratified by quartiles of triglycerides, HDL cholesterol, and TG:HDL

	N	Events	Percent events	Events per 1000 patient-years (95% CI), unadjusted	*p*	Events per 1000 patient-years (95% CI), age- & gender-adjusted	*p*
Triglycerides							
Males					0.507		< 0.0001
I	2219	539	24.3	33.09 (30.41–36.00)	Ref	22.71 (20.70–24.91)	Ref
II	2224	538	24.2	32.75 (30.09–35.64)	0.865	23.36 (21.31–25.61)	0.640
III	212	569	25.7	35.34 (32.56–38.37)	0.273	26.25 (24.01–28.69)	0.016
IV	2247	541	24.1	32.57 (29.94–35.44)	0.796	30.15 (27.63–32.90)	< 0.0001
Females					0.471		0.154
I	1651	330	20.0	26.46 (23.75–29.47)	Ref	18.72 (16.65–21.05)	Ref
II	1710	374	21.9	29.36 (26.53–32.49)	0.167	20.68 (18.49–23.12)	0.188
III	1701	347	20.4	27.11 (24.40–30.11)	0.753	20.27 (18.11–22.70)	0.300
IV	1692	364	21.5	28.75 (25.94–31.86)	0.274	22.25 (19.93–24.84)	0.023
HDL cholesterol							
Males					0.023		< 0.0001
I	2038	542	26.6	36.98 (33.99–40.22)	0.210	32.14 (29.42–35.11)	< 0.0001
II	2200	509	23.1	31.21 (28.61–34.04)	0.120	25.08 (22.88–27.49)	0.304
III	2377	559	23.5	31.70 (29.17–34.44)	0.184	22.76 (20.79–24.91)	0.564
IV	2287	577	25.2	34.30 (31.61–37.21)	Ref	23.55 (21.53–25.76)	Ref
Females					< 0.0001		< 0.0001
I	1654	443	26.8	37.29 (33.98–40.93)	< 0.0001	28.93 (26.12–32.05)	< 0.0001
II	1709	322	18.8	24.79 (22.22–27.65)	0.985	18.77 (16.70–21.09)	0.136
III	176	305	19.4	25.62 (22.90–28.67)	0.685	18.83 (16.72–21.22)	0.131
IV	1815	345	19.0	24.82 (22.33–27.58)	Ref	16.71 (14.88–18.76)	Ref
TG:HDL							
Males					0.656		< 0.0001
I	2223	535	24.1	32.63 (29.97–35.51)	Ref	21.62 (19.69–23.74)	Ref
II	2228	537	24.1	32.64 (30.00–35.53)	0.992	23.38 (21.33–25.62)	0.202
III	2226	565	25.4	34.87 (32.11–37.87)	0.270	26.80 (24.53–29.29)	< 0.0001
IV	2225	550	24.7	33.58 (30.89–36.51)	0.633	30.80 (28.23–33.59)	< 0.0001
Females					0.051		< 0.0001
I	1686	325	19.3	25.31 (22.70–28.22)	Ref	17.33 (15.39–19.52)	Ref
II	1689	347	20.5	27.33 (24.60–30.36)	0.321	19.66 (17.54–22.03)	0.103
III	1690	354	21.0	27.98 (25.22–31.06)	0.191	20.69 (18.49–23.16)	0.022
IV	689	389	23.0	31.15 (28.21–344.1)	0.006	24.41 (21.93–27.17)	< 0.0001

*TG:HDL* triglyceride:HDL cholesterol ratio, *CI* confidence interval

Likewise, no differences among quartiles of triglycerides were detected in Kaplan–Meier estimates (Additional file 2: Figures S1A and S2A) and in unadjusted HRs (not shown) in both males and females. Among male participants, Cox proportional hazards regression analysis showed an increased mortality risk for quartiles III and IV vs quartile I after sequential adjustment for confounders (Additional file 2: Figure S1B-D), including HDL cholesterol levels in case of quartile IV (1.161 [1.019–1.324], *p* = 0.025). Conversely, an increased risk of death was observed among female participants only for quartile IV vs quartile I when adjusted for age, gender and CVD risk factors (Additional file 2: Figure S2B-C), but not when complications/comorbidities (Additional file 2: Figure S2D) and HDL cholesterol levels (0.942 [0.785–1.130], *p* = 0.522) were included in the model.

Crude mortality rates according to quartiles of HDL cholesterol were not different in males, but were significantly higher for quartile I vs quartile IV in females, whereas higher age-adjusted death rates were observed for quartile I vs quartile IV in both males and females (Table 2). Kaplan–Meier estimates (Additional file 2: Figures S3A and S4A) and the unadjusted HRs (not shown) did not differ in males, but were significantly higher for quartile I vs quartile IV in females (1.514 [1.315–1.742], *p* < 0.0001). Likewise, in male participants, a higher risk of death was detected for quartile I vs quartile IV after including age, gender, CVD risk factors, and complications/comorbidities in the model (Additional file 2: Figure S3B-D), but not after further adjustment for triglycerides (1.059 [0.922–1.215], *p* = 0.419). In contrast, in female participants, Cox proportional hazards regression analysis showed a significantly higher risk of death for quartile I vs quartile IV after sequential adjustment for confounders (Additional file 2: Figures S4B-D), including triglyceride levels (1.366 [1.176–1.587], *p* < 0.0001).

Crude mortality rates by quartiles of TG:HDL were not different, whereas age-adjusted death rates were significantly higher for quartiles III and IV vs quartile I in both males and females (Table 2). No differences among quartiles of TG:HDL were observed in Kaplan–Meier estimates (Additional file 2: Figures S5A and S6A) and in unadjusted HRs (not shown) in males, whereas in females unadjusted risk of death was higher for quartile IV vs quartile I (1.234 [1.065–1.430], *p* = 0.005). Cox proportional hazards regression analysis showed a significantly higher risk of death for quartiles III and IV vs quartile I in male participants (Additional file 2: Figure S5B–D) and for quartile IV vs quartile I in female participants (Additional file 2: Figure S6B–D) after adjustment for confounders.

## Discussion

This analysis of patients with type 2 diabetes from the RIACE cohort showed a positive association of triglycerides and TG:HDL and an inverse association of HDL cholesterol with all-cause mortality in a gender-specific manner. The relationship between triglycerides and death was in fact stronger in males, though interaction with gender was not significant, at variance with that of HDL cholesterol, which was a powerful predictor of mortality especially in females.

The independent association of atherogenic dyslipidaemia with all-cause death is consistent with previous results in patients with type 2 diabetes showing a positive relationship with total mortality for triglycerides [32] and TG:HDL [33, 34] and an inverse relationship with CVD morbidity and mortality for HDL cholesterol [23, 24]. In contrast, among Pima Indians with diabetes, an inverse relationship was observed between TG:HDL and all-cause mortality, together with no significant association for triglycerides and U-shaped relationships for total, LDL, and non-HDL cholesterol [17]. However, lower, not higher, concentrations of triglycerides, TG:HDL, and total, LDL, and non-HDL cholesterol were associated with higher total mortality also in individuals without diabetes, suggesting that findings in this population reflect the predominance of causes of death other than CVD [17]. Moreover, Fanni et al. reported no association of HDL cholesterol levels with total, CVD, and cancer mortality and a U-shaped relationship with risk of death from infections in a retrospective population-based cohort study involving 2,113 patients with type 2 diabetes [25]. Likewise, HDL cholesterol levels were not related to CVD events or all-cause mortality in high-risk patients with type 2 diabetes from the Second Manifestations of ARTerial disease cohort [26]. In this study, a positive association was found in individuals with LDL cholesterol < 2.0 mmol/L and an inverse association was observed in those with LDL cholesterol 2.0–2.5 mmol/L (for CVD events only) [26]. However, in the RIACE participants with low LDL cholesterol levels, we confirmed the inverse association between HDL cholesterol and all-cause death detected in the whole cohort.

Altogether, the current and previous findings from the RIACE cohort provide further insights into the understanding of the association of lipid levels with all-cause death by supporting the concept that atherogenic dyslipidaemia characterizing patients with type 2 diabetes plays a major role in the excess mortality of these individuals by conferring an independent risk of death, predominantly by CVD. Moreover, our results indicate that triglyceride and HDL cholesterol levels (and TG:HDL) might perform better than total and LDL cholesterol as predictors of all-cause death in these individuals and that achieving triglyceride and HDL cholesterol goals is particularly important in individuals with type 2 diabetes, despite the fact that current guidelines primarily recommend attaining the LDL cholesterol target with statins and other cholesterol reducing agents [35].

It remains unclear whether the increased mortality risk associated with atherogenic dyslipidaemia is related to the altered circulating levels of triglycerides or HDL cholesterol independent of each other. Concentrations of both triglycerides and HDL cholesterol were shown to be associated with CVD risk, though only HDL cholesterol was an independent predictor of CVD events [36, 37]. In our study, the association of increased triglyceride and reduced HDL cholesterol with all-cause mortality disappeared after adjustment for HDL cholesterol and triglyceride levels, respectively, pointing to common pathophysiological mechanisms. However, this was not the case when men and women were analysed separately (see below). Moreover, it is unclear whether the increased mortality risk associated with atherogenic dyslipidaemia is attributable to increased triglycerides or decreased HDL cholesterol per se or to the related changes in TRLs (and remnant cholesterol content) or HDL function and size, respectively, which however may not be accurately reflected in the lipid profile and might be better predicted by ApoB and ApoA-I, respectively [38]. Regarding CVD morbidity and mortality, Mendelian randomization studies have suggested causality for triglycerides [37], but not for HDL cholesterol [36]. Consistently, intervention trials have shown that fibrates and omega-3 fatty acids, which predominantly reduce triglyceride levels, were effective beyond statin treatment in reducing CVD risk in patients with atherogenic dyslipidaemia [39–43]. In contrast, no additional benefits were observed upon treatment with niacin [44] or cholesteryl ester transfer protein inhibitors [45], which primarily increase HDL cholesterol. On the other hand, previous studies have shown that remnant cholesterol [46] as well as reduced HDL function [47] and increased HDL particle number [48] are associated with CVD risk beyond absolute levels of triglycerides and HDL cholesterol. In addition, the increased all-cause (and CVD) mortality associated with atherogenic dyslipidaemia may be related to the elevated levels of small dense LDLs. These particles are in fact known to be atherogenic because of increased penetration and retention in the vascular wall, due to their reduced size and increased affinity for proteoglycans [49], and decreased affinity for the LDL receptor, due to their enhanced susceptibility to oxidation [50].

A unique observation of our study is the modifying effect of gender, with a predictive role of triglycerides in men and HDL cholesterol in women, which was not attenuated by adjustment for each other, at variance with findings in the whole cohort. Previous gender-specific differences in the association between lipids and mortality were reported only for total cholesterol [11, 18], though not consistently [14, 16], whereas no data exist for abnormalities in lipid fractions characterizing atherogenic dyslipidaemia. Our finding has no obvious explanation and further studies are needed to elucidate the underlying mechanisms. It might reflect differences in lipid metabolism between men and women, which are not entirely explained by differences in sex hormones and require further investigation [51]. In addition, HDL particle characteristics and function may be more important than absolute HDL cholesterol levels in males, as suggested by a cross-sectional study showing that men presented a greater HDL oxidation and lower HDL vasodilatory capacity than women [52]. Our observation has important clinical implications for patients with type 2 diabetes. In fact, risk stratification of these individuals should take into consideration the different impact of triglycerides and HDL cholesterol on mortality in males and females, though it is advisable to achieve the recommended goals for both these lipid fractions regardless of gender. In this view, TG:HDL may be a better predictor than either one for all-cause death and should be preferentially used for estimating mortality risk in people with type 2 diabetes.

Strength of our study include the large sample size, the assessment of a wide range of clinical parameters, and the completeness of baseline and follow-up data. However, there are several limitations. First, the lack of information on the causes of death did not allow to detect possible differences in the relationship of CVD versus non-CVD deaths with lipid levels. Second, lack on information on possible confounders may have affected the results. Unmeasured confounders include levels of inflammatory markers, as inflammation was shown to be independently associated with CVD, heart failure and all-cause mortality regardless of atherogenic lipid levels [53]. Also alcohol consumption was not assessed, though we considered the presence of other comorbidities which may be associated with low lipid levels and high mortality. Moreover, we had no information on lipid profile and lipid-lowering treatments over time. Since only 46.2% of participants were on lipid-lowering treatment at baseline, this percentage has probably increased during the follow-up period, with consequent changes in lipid levels. However, most of these individuals (42.5%) were on statins, which affect mainly LDL cholesterol levels, with very few patients receiving other lipid-lowering agents (omega-3, 4.9%, fibrates, 2.5%, ezetimibe, 1.0%, and resins, 0.1%) and a negligible percentage on dual (4.4%) or triple (0.2%) therapy. Third, the study findings may not be applicable to the general ambulatory population, as only part of the individuals with type 2 diabetes attend Diabetes Clinics in Italy. Finally, the observational design makes causal interpretation impossible.

## Conclusion

In patients with type 2 diabetes, atherogenic dyslipidaemia was independently associated with all-cause death, at variance with total or LDL cholesterol. There was a stronger impact on mortality of triglycerides in males and HDL cholesterol in females, suggesting that TG:HDL may be the best lipid marker for predicting death from any cause in these individuals.

## Supplementary Information

**Additional file 1: Table S1.** Baseline clinical features of study participants by quartiles of triglycerides. **Table S2:** Baseline clinical features of study participants by quartiles of HDL cholesterol. **Table S3:** Baseline clinical features of study participants by quartiles of TG:HDL.

**Additional file 2: Figure S1.** Survival analysis by quartiles of triglycerides in males. **Figure S2.** Survival analysis by quartiles of triglycerides in females. **Figure S3.** Survival analysis by quartiles of HDL cholesterol in males. **Figure S4.** Survival analysis by quartiles of HDL cholesterol in females. **Figure S5.** Survival analysis by quartiles of TG:HDL in males. **Figure S6.** Survival analysis by quartiles of TG:HDL in females.

**Additional file 3.** The RIACE Study Group

## Data Availability

The datasets used and/or analysed during the current study are available from the corresponding author on reasonable request.

## Abbreviations

CVD: Cardiovascular disease
Apo: Apolipoprotein
TRLs: Triglycerides-rich lipoproteins
RIACE: Renal Insufficiency and Cardiovascular Events
eGFR: Estimated glomerular filtration rate
BP: Blood pressure
BMI: Body mass index
HbA_1c_: Haemoglobin A_1c_
TG:HDL: Triglyceride:HDL cholesterol ratio
DKD: Diabetic kidney disease
DR: Diabetic retinopathy
CI: Confidence interval
HR: Hazard ratio
