# Supplementary material for: Independent association of atherogenic dyslipidaemia with all‐cause mortality in individuals with type 2 diabetes and modifying effect of gender: a prospective cohort study
Source: Cardiovasc Diabetol. 2021 Jan 30;20:28. doi: 10.1186/s12933-021-01224-7 (PMC7847015; doi:10.1186/s12933-021-01224-7)
Supplement: Supplementary file 1 — Additional file 1: Table S1. Baseline clinical features of study participants by quartiles of triglycerides. Table S2: Baseline clinical features of study participants by quartiles of HDL cholesterol. Table S3: Baseline clinical features of study participants by quartiles of TG:HDL. [file 12933_2021_1224_MOESM1_ESM.doc]

**Additional file 1 Additional Tables**

**Table S1.** Baseline clinical features of study participants by quartiles of triglycerides.

| **Variables** | **l** | **II** | **III** | **IV** | ***p*** |
| --- | --- | --- | --- | --- | --- |
| **N (%)** | 3,992 (25.5) | 3,839 (24.5) | 3,933 (25.1) | 3,892 (24.9) |  |
| **Triglycerides, mmol·l^-1^** | 0.77±0.14 | 1.16±0.10 | 1.59±0.16 | 2.80±1.26 |  |
|  | (0.23-0.97) | (0.98-1.33) | (1.34-1.89) | (1.89-21.96) |  |
| **Deaths, n (%)** | 897 (22.5) | 886 (23.1) | 929 (23.6) | 890 (22.9) | 0.673 |
| **Age, years** | 67.2±10.6 | 67.3±10.2 | 66.9±10.0 | 65.0±10.3 | <0.0001 |
| **Gender, n (%)** |  |  |  |  | <0.0001 |
| **Females** | 1,531 (38.4) | 1,741 (45.4) | 1,811 (46.0) | 1,671 (42.9) |  |
| **Males** | 2,461 (61.6) | 2,098 (54.6) | 2,122 (54.0) | 2,221 (57.1) |  |
| **Smoking, n (%)** |  |  |  |  | <0.0001 |
| **Never** | 2,348 (58.8) | 2,249 (58.6) | 2,251 (57.2) | 2,001 (51.4) |  |
| **Former** | 1,079 (27.0) | 1,063 (27.7) | 1,084 (27.6) | 1,181 (30.3) |  |
| **Current** | 565 (14.2) | 527 (13.7) | 598 (15.2) | 710 (18.2) |  |
| **Diabetes duration, years** | 14.7±10.8 | 13.3±10.3 | 12.6±9.8 | 12.1±9.5 | <0.0001 |
| **HbA_1c_, mmol·mol^-1^** | 56.0±14.2 | 57.8±15.2 | 58.8±16.3 | 63.3±18.8 | <0.0001 |
| **BMI, kg·m^-2^** | 27.3±4.8 | 28.7±5.2 | 29.4±5.1 | 30.4±5.1 | <0.0001 |
| **Waist circumference, cm** | 99.1±9.7 | 102.0±10.4 | 103.4±10.1 | 105.5±10.3 | <0.0001 |
| **Total cholesterol, mmol·l^-1^** | 4.45±0.87 | 4.69±0.92 | 4.82±0.93 | 5.18±1.09 | <0.0001 |
| **HDL cholesterol, mmol·l^-1^** | 1.47±0.38 | 1.34±0.34 | 1.24±0.31 | 1.12±0.28 | <0.0001 |
| **TG:HDL ratio** | 1.29±0.46 | 2.11±0.60 | 3.13±0.88 | 6.23±3.90 | <0.0001 |
| **Non-HDL cholesterol, mmol·l^-1^** | 2.99±0.76 | 3.35±0.82 | 3.58±0.85 | 4.06±1.02 | <0.0001 |
| **LDL cholesterol, mmol·l^-1^** | 2.64±0.75 | 2.82±0.81 | 2.86±0.85 | 2.84±0.95 | <0.0001 |
| **Dyslipidaemia, n (%)** | 2,974 (74.5) | 3,196 (83.3) | 3,346 (85.1) | 3,340 (85.8) | <0.0001 |
| **Systolic BP, mmHg** | 136.8±18.1 | 138.0±17.6 | 138.9±17.9 | 138.5±18.4 | <0.0001 |
| **Diastolic BP, mmHg** | 78.0±9.2 | 78.7±9.2 | 78.9±9.3 | 79.5±9.9 | <0.0001 |
| **Pulse pressure, mmHg** | 58.8±15.6 | 59.3±15.5 | 60.0±16.1 | 59.1±15.6 | 0.005 |
| **Hypertension, n (%)** | 3,148 (78.9) | 3,211 (83.6) | 3,369 (85.7) | 3,368 (86.5) | <0.0001 |
| **Anti-hyperglycaemic treatment, n (%)** |  |  |  |  | <0.0001 |
| **Lifestyle** | 616 (15.4) | 524 (13.6) | 492 (12.5) | 481 (12.4) |  |
| **Non-insulin** | 2,354 (59.0) | 2,419 (63.0) | 2,496 (63.5) | 2,350 (60.4) |  |
| **Insulin** | 1,022 (25.6) | 896 (23.3) | 945 (24.0) | 1,061 (27.3) |  |
| **Lipid-lowering treatment, n (%)** | 1,552 (38.9) | 1,668 (43.4) | 1,904 (48.4) | 2,114 (54.3) | <0.0001 |
| **Anti-hypertensive treatment, n (%)** | 2,556 (64.0) | 2,725 (71.0) | 2,892 (73.5) | 2,899 (74.5) | <0.0001 |
| **Anti-platelet treatment, n (%)** | 1,492 (37.4) | 1,518 (39.5) | 1,634 (41.5) | 1,604 (41.2) | <0.0001 |
| **Anti-coagulant treatment, n (%)** | 154 (3.9) | 185 (4.8) | 167 (4.2) | 163 (4.2) | 0.207 |
| **Albuminuria, mg·day^-1^** | 50.2±327.3 | 56.8±222.9 | 69.9±255.0 | 112.8±421.1 | <0.0001 |
| **Serum creatinine, μmol·l^-1^** | 76.0±25.6 | 78.7±28.3 | 82.2±39.8 | 86.6±40.7 | <0.0001 |
| **eGFR, ml·min^-1^·1.73m^-2^** | 83.5±18.5 | 80.8±19.9 | 79.2±21.1 | 77.5±23.5 | <0.0001 |
| **DKD phenotype, n (%)** |  |  |  |  | <0.0001 |
| **No DKD** | 2,868 (71.8) | 2,550 (66.4) | 2,445 (62.2) | 2,121 (54.5) |  |
| **Albuminuric DKD with preserved eGFR** | 671 (16.8) | 678 (17.7) | 748 (19.0) | 869 (22.3) |  |
| **Nonalbuminuric DKD** | 275 (6.9) | 365 (9.5) | 392 (10.0) | 444 (11.4) |  |
| **Albuminuric DKD with reduced eGFR** | 178 (4.5) | 246 (6.4) | 348 (8.8) | 458 (11.8) |  |
| **DR, n (%)** |  |  |  |  | 0.001 |
| **No DR** | 3,075 (77.0) | 2,991 (77.9) | 3,107 (79.0) | 3,016 (77.5) |  |
| **Non-advanced DR** | 564 (14.1) | 480 (12.5) | 432 (11.0) | 471 (12.1) |  |
| **Advanced DR** | 353 (8.8) | 368 (9.6) | 394 (10.0) | 405 (10.4) |  |
| **CVD, n (%)** |  |  |  |  |  |
| **Any** | 847 (21.2) | 882 (23.0) | 939 (23.9) | 952 (24.5) | 0.004 |
| **Myocardial infarction** | 386 (9.7) | 399 (10.4) | 474 (12.1) | 483 (12.4) | <0.0001 |
| **Coronary revascularization** | 353 (8.8) | 384 (10.0) | 410 (10.4) | 432 (11.1) | 0.008 |
| **Any coronary event** | 540 (13.5) | 577 (15.0) | 632 (16.1) | 647 (16.6) | 0.001 |
| **Stroke** | 112 (2.8) | 134 (3.5) | 127 (3.2) | 140 (3.6) | 0.200 |
| **Carotid revascularization** | 204 (5.1) | 220 (5.7) | 208 (5.3) | 224 (5.8) | 0.500 |
| **Any carotid event** | 301 (7.5) | 331 (8.6) | 317 (8.1) | 343 (8.8) | 0.159 |
| **Ulcer/gangrene/amputation** | 124 (3.1) | 149 (3.9) | 134 (3.4) | 149 (3.8) | 0.200 |
| **Lower limb revascularization** | 94 (2.4) | 113 (2.9) | 112 (2.8) | 131 (3.4) | 0.063 |
| **Any peripheral event** | 193 (4.8) | 225 (5.9) | 219 (5.6) | 246 (6.3) | 0.034 |
| **Comorbidities n (%)** |  |  |  |  |  |
| **Any** | 685 (17.2) | 706 (18.4) | 712 (18.1) | 684 (17.6) | 0.491 |
| **COPD** | 134 (3.4) | 178 (4.6) | 187 (4.8) | 175 (4.5) | 0.007 |
| **Chronic liver disease** | 403 (10.1) | 334 (8.7) | 320 (8.1) | 304 (7.8) | 0.002 |
| **Cancer** | 219 (5.5) | 253 (6.6) | 277 (7.0) | 282 (7.2) | 0.008 |

HbA_1c_ = haemoglobin A_1c_; BMI = body mass index; TG:HDL = triglyceride:HDL cholesterol ratio; BP = blood pressure; eGFR = estimated glomerular filtration rate; DKD = diabetic kidney disease; DR = diabetic retinopathy; = CVD = cardiovascular disease; COPD = chronic obstructive pulmonary disease.

**Table S2.** Baseline clinical features of study participants by quartiles of HDL cholesterol.

| **Variables** | **l** | **II** | **III** | **IV** | ***p*** |
| --- | --- | --- | --- | --- | --- |
| **N (%)** | 4,025 (25.7) | 3,982 (25.4) | 3,769 (24.1) | 3,880 (24.8) |  |
| **HDL cholesterol, mmol·l^-1^** | 0.90±0.12 | 1.15±0.06 | 1.36±0.07 | 1.77±0.27 |  |
|  | (0.26-1.04) | (1.04-1.24) | (1.25-1.48) | (1.48-3.70) |  |
| **Deaths, n (%)** | 1,061 (26.4) | 881 (22.1) | 818 (21.7) | 842 (21.7) | <0.0001 |
| **Age, years** | 65.0±10.7 | 66.1±10.2 | 67.2±10.1 | 68.2±10.0 | <0.0001 |
| **Gender, n (%)** |  |  |  |  | <0.0001 |
| **Females** | 1,101 (27.4) | 1,549 (38.9) | 1,803 (47.8) | 2,301 (59.3) |  |
| **Males** | 2,924 (72.6) | 2,433 (61.1) | 1,966 (52.2) | 1,579 (40.7) |  |
| **Smoking, n (%)** |  |  |  |  | <0.0001 |
| **Never** | 1,903 (47.3) | 2,188 (54.9) | 2,256 (59.9) | 2,502 (64.5) |  |
| **Former** | 1,307 (32.5) | 1,148 (28.8) | 995 (26.4) | 957 (24.7) |  |
| **Current** | 815 (20.2) | 646 (16.2) | 518 (13.7) | 421 (10.9) |  |
| **Diabetes duration, years** | 12.3±9.8 | 13.0±10.0 | 13.3±10.3 | 14.2±10.5 | <0.0001 |
| **HbA_1c_, mmol·mol^-1^** | 60.8±17.8 | 59.3±16.4 | 58.4±16.1 | 57.4±15.1 | <0.0001 |
| **BMI, kg·m^-2^** | 29.7±5.0 | 29.3±5.1 | 28.9±5.0 | 27.9±5.2 | <0.0001 |
| **Waist circumference, cm** | 104.4±10.3 | 103.3±10.4 | 102.2±10.0 | 100.0±10.4 | <0.0001 |
| **Triglycerides, mmol·l^-1^** | 2.05±1.33 | 1.64±0.87 | 1.42±0.72 | 1.17±0.66 | <0.0001 |
| **Total cholesterol, mmol·l^-1^** | 4.40±0.99 | 4.73±0.95 | 4.89±0.94 | 5.13±0.93 | <0.0001 |
| **TG:HDL ratio** | 5.39±4.03 | 3.28±1.78 | 2.39±1.23 | 1.55±0.94 |  |
| **Non-HDL cholesterol, mmol·l^-1^** | 3.50±0.98 | 3.58±0.95 | 3.53±0.93 | 3.36±0.93 | <0.0001 |
| **LDL cholesterol, mmol·l^-1^** | 2.60±0.83 | 2.84±0.83 | 2.88±0.84 | 2.83±0.85 | <0.0001 |
| **Dyslipidaemia, n (%)** | 3,160 (78.5) | 3,349 (84.1) | 3,197 (84.8) | 3,150 (81.2) | <0.0001 |
| **Systolic BP, mmHg** | 136.6±18.1 | 137.8±17.9 | 138.8±17.8 | 139.2±18.1 | <0.0001 |
| **Diastolic BP, mmHg** | 78.6±9.7 | 78.8±9.4 | 79.1±9.3 | 78.5±9.3 | 0.044 |
| **Pulse pressure, mmHg** | 12.3±9.8 | 13.0±10.0 | 13.3±10.3 | 14.2±10.5 | 0.874 |
| **Hypertension, n (%)** | 3,416 (84.9) | 3,355 (84.3) | 3,167 (84.0) | 3,158 (81.4) | 0.001 |
| **Anti-hyperglycaemic treatment, n (%)** |  |  |  |  | <0.0001 |
| **Lifestyle** | 483 (12.0) | 527 (13.2) | 529 (14.0) | 574 (14.8) |  |
| **Non-insulin** | 2,451 (60.9) | 2,517 (63.2) | 2,421 (64.2) | 2,230 (57.5) |  |
| **Insulin** | 1,091 (27.1) | 938 (23.6) | 819 (21.7) | 1,076 (27.7) |  |
| **Lipid-lowering treatment, n (%)** | 1,920 (47.7) | 1,888 (47.4) | 1,728 (45.8) | 1,702 (43.9) | 0.002 |
| **Anti-hypertensive treatment, n (%)** | 2,979 (74.0) | 2,880 (72.3) | 2,664 (70.7) | 2,549 (65.7) | <0.0001 |
| **Anti-platelet treatment, n (%)** | 1,701 (42.3) | 1,657 (41.6) | 1,451 (38.5) | 1,439 (37.1) | <0.0001 |
| **Anti-coagulant treatment, n (%)** | 250 (6.2) | 155 (3.9) | 133 (3.5) | 131 (3.4) | <0.0001 |
| **Albuminuria, mg·day^-1^** | 90.8±290.1 | 67.0±287.0 | 65.5±278.1 | 65.2±397.6 | <0.0001 |
| **Serum creatinine, μmol·l^-1^** | 88.4±40.7 | 82.2±33.6 | 77.8±27.4 | 76.0±32.7 | <0.0001 |
| **eGFR, ml·min^-1^·1.73m^-2^** | 78.6±23.0 | 80.2±21.2 | 81.2±19.3 | 81.2±19.7 | <0.0001 |
| **DKD phenotype, n (%)** |  |  |  |  | <0.0001 |
| **No DKD** | 2,228 (55.4) | 2,526 (63.4) | 2,524 (67.0) | 2,706 (69.7) |  |
| **Albuminuric DKD with preserved eGFR** | 927 (23.0) | 755 (19.0) | 677 (18.0) | 607 (15.6) |  |
| **Nonalbuminuric DKD** | 411 (10.2) | 390 (9.8) | 342 (9.1) | 333 (8.6) |  |
| **Albuminuric DKD with reduced eGFR** | 459 (11.4) | 311 (7.8) | 226 (6.0) | 234 (6.0) |  |
| **DR, n (%)** |  |  |  |  | 0.028 |
| **No DR** | 3,075 (76.4) | 3,132 (78.7) | 2,964 (78.6) | 3,018 (77.8) |  |
| **Non-advanced DR** | 505 (12.5) | 474 (11.9) | 472 (12.5) | 496 (12.8) |  |
| **Advanced DR** | 445 (11.1) | 376 (9.4) | 333 (8.8) | 366 (9.4) |  |
| **CVD, n (%)** |  |  |  |  |  |
| **Any** | 1,131 (28.1) | 998 (25.1) | 776 (20.6) | 715 (18.4) | <0.0001 |
| **Myocardial infarction** | 594 (14.8) | 457 (11.5) | 367 (9.7) | 324 (8.4) | <0.0001 |
| **Coronary revascularization** | 540 (13.4) | 432 (10.8) | 338 (9.0) | 269 (6.9) | <0.0001 |
| **Any coronary event** | 789 (19.6) | 644 (16.2) | 519 (13.8) | 444 (11.4) | <0.0001 |
| **Stroke** | 148 (3.7) | 139 (3.5) | 114 (3.0) | 112 (2.9) | 0.156 |
| **Carotid revascularization** | 247 (6.1) | 267 (6.7) | 187 (5.0) | 155 (4.0) | <0.0001 |
| **Any carotid event** | 369 (9.2) | 382 (9.6) | 285 (7.6) | 256 (6.6) | <0.0001 |
| **Ulcer/gangrene/amputation** | 192 (4.8) | 143 (3.6) | 110 (2.9) | 111 (2.9) | <0.0001 |
| **Lower limb revascularization** | 162 (4.0) | 128 (3.2) | 85 (2.3) | 75 (1.9) | <0.0001 |
| **Any peripheral event** | 308 (7.7) | 239 (6.0) | 170 (4.5) | 166 (4.3) | <0.0001 |
| **Comorbidities n (%)** |  |  |  |  |  |
| **Any** | 800 (17.4) | 692 (16.6) | 625 (19.9) | 670 (17.3) | 0.001 |
| **COPD** | 207 (5.1) | 158 (4.0) | 134 (3.6) | 175 (4.5) | 0.004 |
| **Chronic liver disease** | 385 (9.6) | 343 (8.6) | 312 (8.3) | 321 (8.3) | 0.135 |
| **Cancer** | 304 (7.6) | 248 (6.2) | 247 (6.6) | 232 (6.0) | 0.026 |

HbA_1c_ = haemoglobin A_1c_; BMI = body mass index; TG:HDL = triglyceride:HDL cholesterol ratio; BP = blood pressure; eGFR = estimated glomerular filtration rate; DKD = diabetic kidney disease; DR = diabetic retinopathy; = CVD = cardiovascular disease; COPD = chronic obstructive pulmonary disease.

**Table S3.** Baseline clinical features of study participants by TG:HDL.

| **Variables** | **I** | **II** | **III** | **IV** | ***p*** |
| --- | --- | --- | --- | --- | --- |
| **N (%)** | 3,914 (22.1) | 3,916 (22.2) | 3,915 (23.7) | 3,911 (24.1) |  |
| **TG:HDL ratio** | 1.17±0.29 | 2.03±0.26 | 3.09±0.40 | 6.44±3.76 |  |
|  | (0.27-1.60) | (1.60-2.49) | (2.49-3.85) | (3.85-85.06) |  |
| **Deaths, n (%)** | 863 (22.0) | 869 (22.2) | 927 (23.7) | 943 (24.1) | 0.066 |
| **Age, years** | 67.8±10.3 | 67.1±10.2 | 66.6±10.2 | 64.8±10.4 | <0.0001 |
| **Gender, n (%)** |  |  |  |  | <0.0001 |
| **Females** | 1,829 (46.7) | 1,759 (44.9) | 1,724 (44.0) | 1,442 (36.9) |  |
| **Males** | 2,085 (53.3) | 2,157 (55.1) | 2,191 (56.0) | 2,469 (63.1) |  |
| **Smoking, n (%)** |  |  |  |  | <0.0001 |
| **Never** | 2,431 (62.1) | 2,332 (59.6) | 2,150 (54.9) | 1,936 (49.5) |  |
| **Former** | 1,013 (25.9) | 1,046 (26.7) | 1,120 (28.6) | 1,228 (31.4) |  |
| **Current** | 470 (12.0) | 538 (13.7) | 645 (16.5) | 747 (19.1) |  |
| **Diabetes duration, years** | 14.8±10.8 | 13.2±10.3 | 12.7±9.9 | 12.1±9.6 | <0.0001 |
| **HbA_1c_, mmol·mol^-1^** | 56.3±14.2 | 57.3±15.1 | 59.2±16.4 | 63.1±18.9 | <0.0001 |
| **BMI, kg·m^-2^** | 27.2±4.8 | 28.9±5.1 | 29.5±5.1 | 30.3±5.0 | <0.0001 |
| **Waist circumference, cm** | 98.8±9.8 | 102.2±10.3 | 103.5±10.3 | 105.4±10.2 | <0.0001 |
| **Triglycerides, mmol·l^-1^** | 0.81±0.21 | 1.18±0.24 | 1.59±0.33 | 2.71±1.31 | <0.0001 |
| **Total cholesterol, mmol·l^-1^** | 4.71±0.89 | 4.70±0.94 | 4.77±0.98 | 4.95±1.12 | <0.0001 |
| **HDL cholesterol, mmol·l^-1^** | 1.63±0.35 | 1.34±0.25 | 1.18±0.23 | 1.00±0.21 | <0.0001 |
| **Non-HDL cholesterol, mmol·l^-1^** | 3.07±0.79 | 3.36±0.84 | 3.59±0.89 | 3.95±1.04 | <0.0001 |
| **LDL cholesterol, mmol·l^-1^** | 2.70±0.77 | 2.82±0.82 | 2.86±0.85 | 2.77±0.93 | <0.0001 |
| **Dyslipidaemia, n (%)** | 3,003 (76.7) | 3,253 (83.1) | 3,325 (84.9) | 3,275 (83.7) | <0.0001 |
| **Systolic BP, mmHg** | 137.8±18.0 | 137.9±17.8 | 138.5±17.8 | 138.0±18.4 | 0.315 |
| **Diastolic BP, mmHg** | 78.2±9.1 | 78.6±9.3 | 79.1±9.3 | 79.2±10.0 | <0.0001 |
| **Pulse pressure, mmHg** | 59.6±15.7 | 59.3±15.6 | 59.5±15.8 | 58.8±15.7 | 0.132 |
| **Hypertension, n (%)** | 3,103 (79.3) | 3,281 (83.8) | 3,352 (85.6) | 3,360 (85.9) | <0.0001 |
| **Anti-hyperglycaemic treatment, n (%)** |  |  |  |  | <0.0001 |
| **Lifestyle** | 610 (15.6) | 533 (13.6) | 492 (12.6) | 478 (12.2) |  |
| **Non-insulin** | 2,252 (57.5) | 2,496 (63.7) | 2,542 (64.9) | 2,329 (59.5) |  |
| **Insulin** | 1,052 (26.9) | 887 (22.7) | 881 (22.5) | 1,104 (28.2) |  |
| **Lipid-lowering treatment, n (%)** | 1,553 (39.7) | 1,737 (44.4) | 1,871 (47.8) | 2,077 (53.1) | <0.0001 |
| **Anti-hypertensive treatment, n (%)** | 2,489 (63.6) | 2,784 (71.1) | 2,890 (73.8) | 2,909 (74.4) | <0.0001 |
| **Anti-platelet treatment, n (%)** | 1,422 (36.3) | 1,581 (40.4) | 1,608 (41.1) | 1,637 (41.9) | <0.0001 |
| **Anti-coagulant treatment, n (%)** | 136 (3.5) | 170 (4.3) | 178 (4.5) | 185 (4.7) | 0.031 |
| **Albuminuria, mg·day^-1^** | 52.8±340.9 | 55.4±210.2 | 73.0±284.3 | 108.1±398.7 | <0.0001 |
| **Serum creatinine, μmol·l^-1^** | 75.1±26.5 | 78.7±29.2 | 82.2±34.5 | 88.4±43.3 | <0.0001 |
| **eGFR, ml·min^-1^·1.73m^-2^** | 82.9±18.6 | 81.3±19.6 | 79.5±21.3 | 77.4±23.6 | <0.0001 |
| **DKD phenotype, n (%)** |  |  |  |  | <0.0001 |
| **No DKD** | 2,828 (72.3) | 2,646 (67.6) | 2,403 (61.4) | 2,107 (53.9) |  |
| **Albuminuric DKD with preserved eGFR** | 621 (15.9) | 685 (17.5) | 765 (19.5) | 895 (22.9) |  |
| **Nonalbuminuric DKD** | 287 (7.3) | 346 (8.8) | 409 (10.4) | 434 (11.1) |  |
| **Albuminuric DKD with reduced eGFR** | 178 (4.5) | 239 (6.1) | 338 (8.6) | 475 (12.1) |  |
| **DR, n (%)** |  |  |  |  | <0.0001 |
| **No DR** | 3,036 (77.6) | 3,050 (77.9) | 3,072 (78.5) | 3,031 (77.5) |  |
| **Non-advanced DR** | 549 (14.0) | 494 (12.6) | 440 (11.2) | 464 (11.9) |  |
| **Advanced DR** | 329 (8.4) | 372 (9.5) | 403 (10.3) | 416 (10.6) |  |
| **CVD, n (%)** |  |  |  |  |  |
| **Any** | 780 (19.9) | 860 (22.0) | 962 (24.6) | 1,018 (26.0) | <0.0001 |
| **Myocardial infarction** | 351 (9.0) | 391 (10.0) | 469 (12.0) | 531 (13.6) | <0.0001 |
| **Coronary revascularization** | 311 (7.9) | 370 (9.4) | 424 (10.8) | 474 (12.1) | <0.0001 |
| **Any coronary event** | 490 (12.5) | 565 (14.4) | 638 (16.3) | 703 (18.0) | <0.0001 |
| **Stroke** | 110 (2.8) | 124 (3.2) | 144 (3.7) | 135 (3.5) | 0.157 |
| **Carotid revascularization** | 182 (4.6) | 219 (5.6) | 224 (5.7) | 231 (5.9) | 0.068 |
| **Any carotid event** | 278 (7.1) | 321 (8.2) | 348 (8.9) | 345 (8.8) | 0.014 |
| **Ulcer/gangrene/amputation** | 114 (2.9) | 138 (3.5) | 142 (3.6) | 162 (4.1) | 0.033 |
| **Lower limb revascularization** | 82 (2.1) | 101 (2.6) | 116 (3.0) | 151 (3.9) | <0.0001 |
| **Any peripheral event** | 175 (4.5) | 206 (5.3) | 224 (5.7) | 278 (7.1) | <0.0001 |
| **Comorbidities n (%)** |  |  |  |  |  |
| **Any** | 667 (17.0) | 692 (17.7) | 701 (17.9) | 727 (18.6) | 0.351 |
| **COPD** | 135 (3.4) | 164 (4.2) | 186 (4.8) | 189 (4.8) | 0.009 |
| **Chronic liver disease** | 365 (9.3) | 351 (9.0) | 308 (7.9) | 337 (8.6) | 0.126 |
| **Cancer** | 226 (5.8) | 242 (6.2) | 273 (7.0) | 290 (7.4) | 0.014 |

TG:HDL = triglyceride:HDL cholesterol ratio; HbA_1c_ = haemoglobin A_1c_; BMI = body mass index; BP = blood pressure; eGFR = estimated glomerular filtration rate; DKD = diabetic kidney disease; DR = diabetic retinopathy; = CVD = cardiovascular disease; COPD = chronic obstructive pulmonary disease.
