# Supplementary material for: Independent association of atherogenic dyslipidaemia with all‐cause mortality in individuals with type 2 diabetes and modifying effect of gender: a prospective cohort study
Source: Cardiovasc Diabetol. 2021 Jan 30;20:28. doi: 10.1186/s12933-021-01224-7 (PMC7847015; doi:10.1186/s12933-021-01224-7)
Supplement: Supplementary file 2 — Additional file 2: Figure S1. Survival analysis by quartiles of triglycerides in males. Figure S2. Survival analysis by quartiles of triglycerides in females. Figure S3. Survival analysis by quartiles of HDL cholesterol in males. Figure S4. Survival analysis by quartiles of HDL cholesterol in females. Figure S5. Survival analysis by quartiles of TG:HDL in males. Figure S6. Survival analysis by quartiles of TG:HDL in females. [file 12933_2021_1224_MOESM2_ESM.doc]

**Additional file 2: Additional Figures**

**
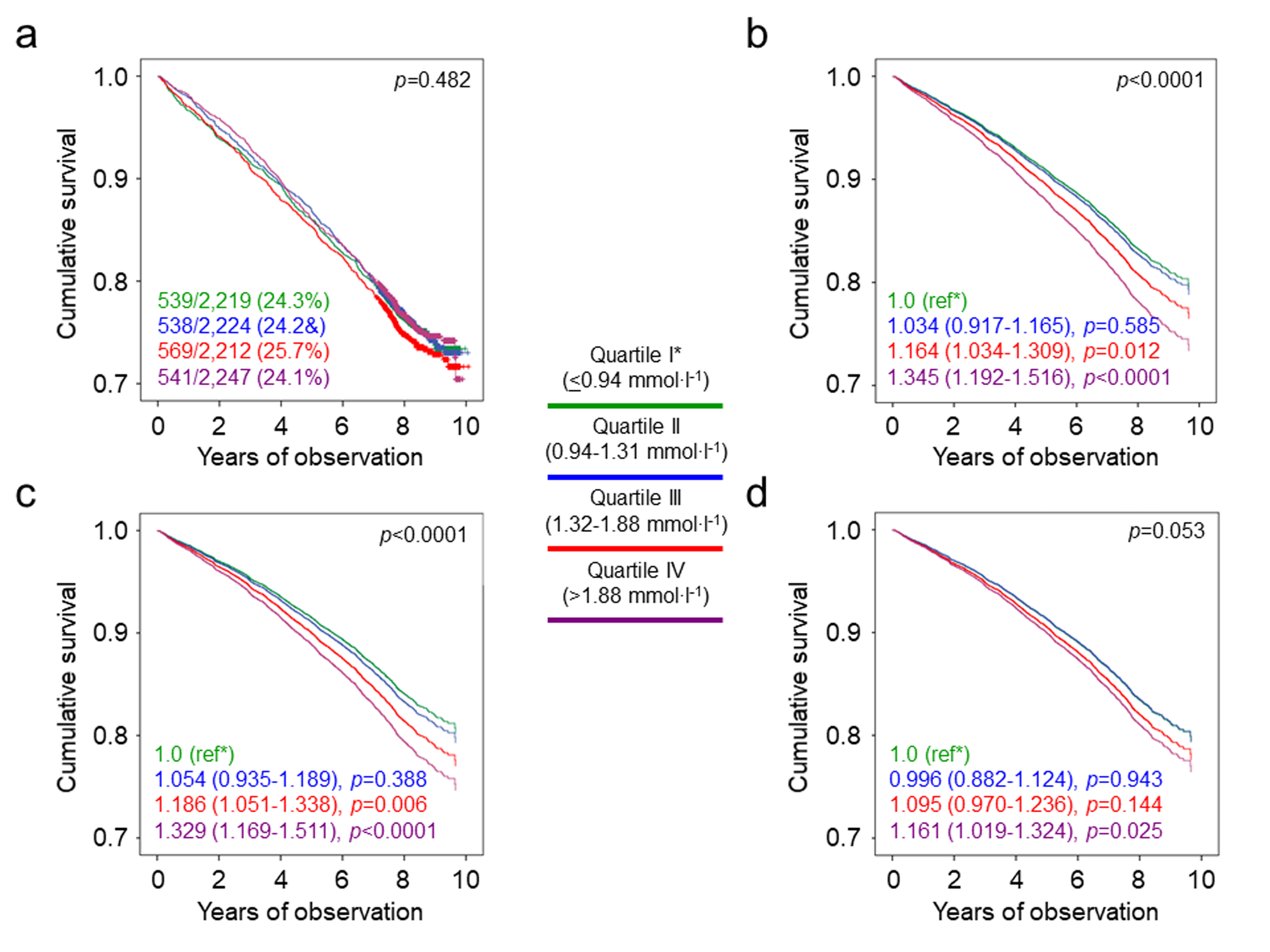
**

**Figure S1.** Survival analysis by quartiles of triglycerides in males. Cumulative survival by Kaplan Meier-analysis (a) and Cox proportional hazards regression, adjusted for age and gender (b), plus CVD risk factors (c) plus complications/comorbidities (d), according to quartiles of triglycerides in males. Numbers (percentages) of deaths and HRs (95% CI) for mortality are shown for each group. HR = hazard ratio; CI = confidence interval.

**
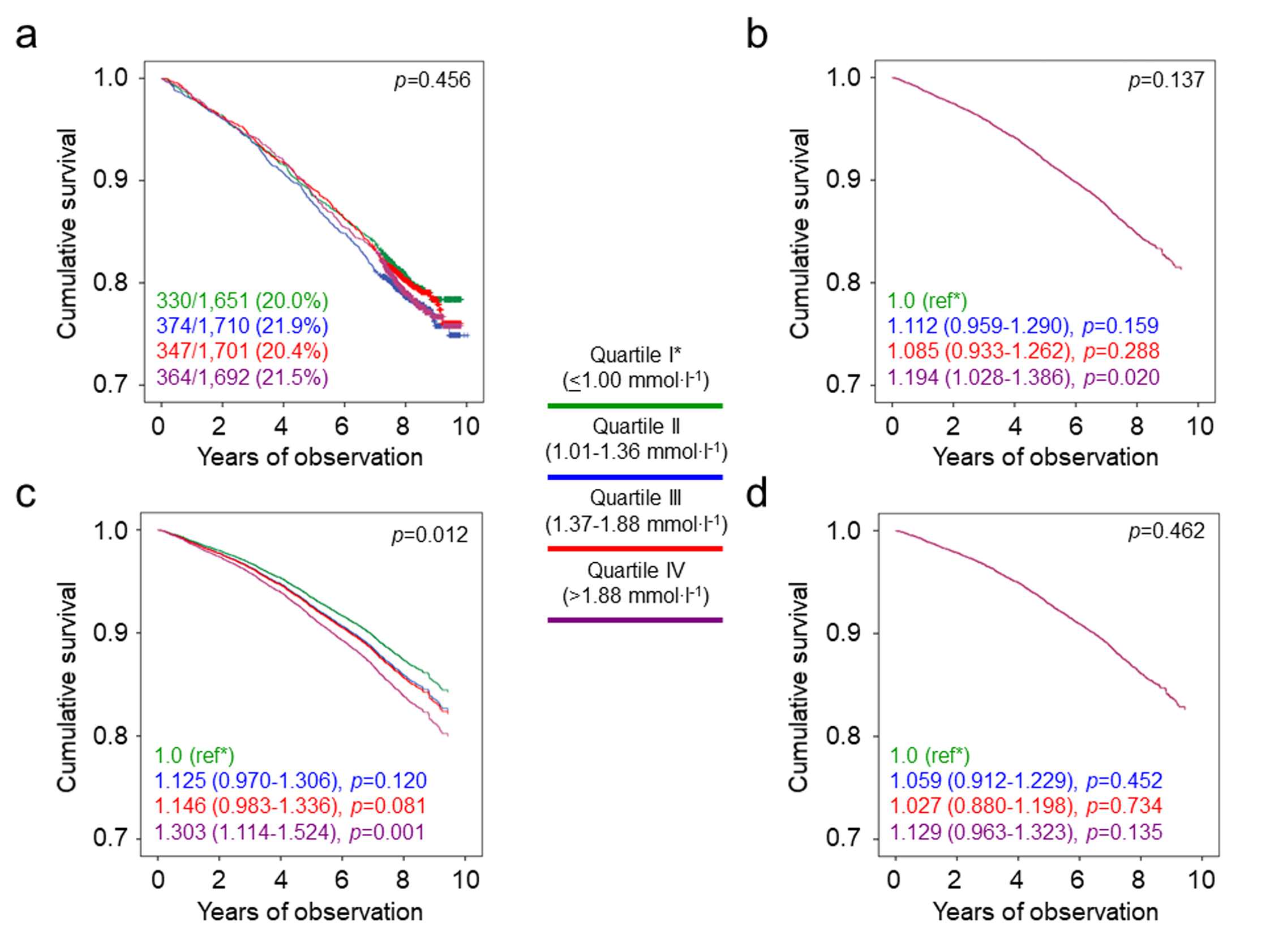
**

**Figure S2.** Survival analysis by quartiles of triglycerides in females. Cumulative survival by Kaplan Meier-analysis (a) and Cox proportional hazards regression, adjusted for age and gender (b), plus CVD risk factors (c) plus complications/comorbidities (d), according to quartiles of triglycerides in females. Numbers (percentages) of deaths and HRs (95% CI) for mortality are shown for each group. HR = hazard ratio; CI = confidence interval.

**
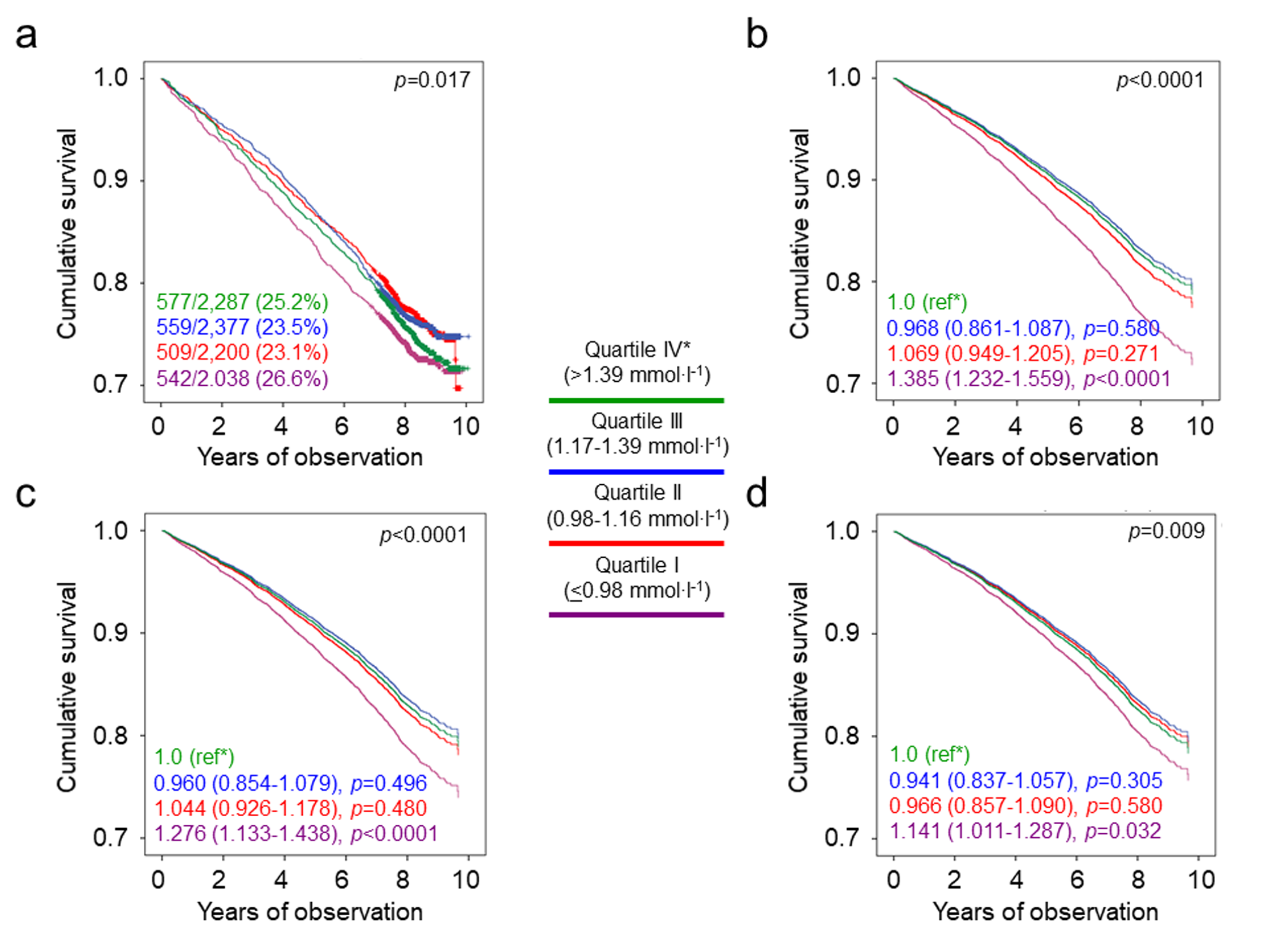
**

**Figure S3.** Survival analysis by quartiles of HDL cholesterol in males. Cumulative survival by Kaplan Meier-analysis (a) and Cox proportional hazards regression, adjusted for age and gender (b), plus CVD risk factors (c) plus complications/comorbidities (d), according to quartiles of HDL cholesterol in males. Numbers (percentages) of deaths and HRs (95% CI) for mortality are shown for each group. HR = hazard ratio; CI = confidence interval.

**
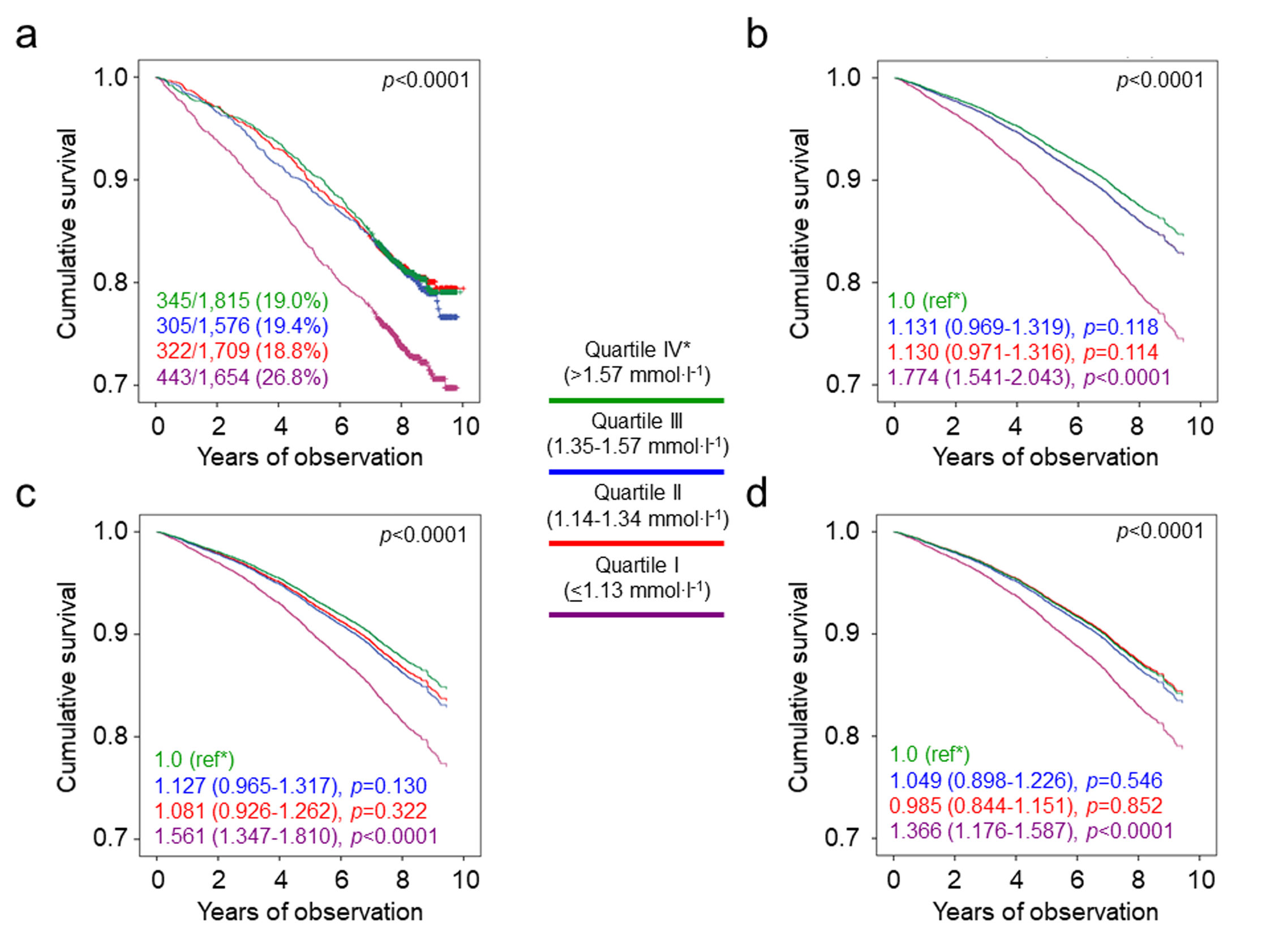
**

**Figure S4.** Survival analysis by quartiles of HDL cholesterol in females. Cumulative survival by Kaplan Meier-analysis (a) and Cox proportional hazards regression, adjusted for age and gender (b), plus CVD risk factors (c) plus complications/comorbidities (d), according to quartiles of HDL cholesterol in females. Numbers (percentages) of deaths and HRs (95% CI) for mortality are shown for each group. HR = hazard ratio; CI = confidence interval.

**
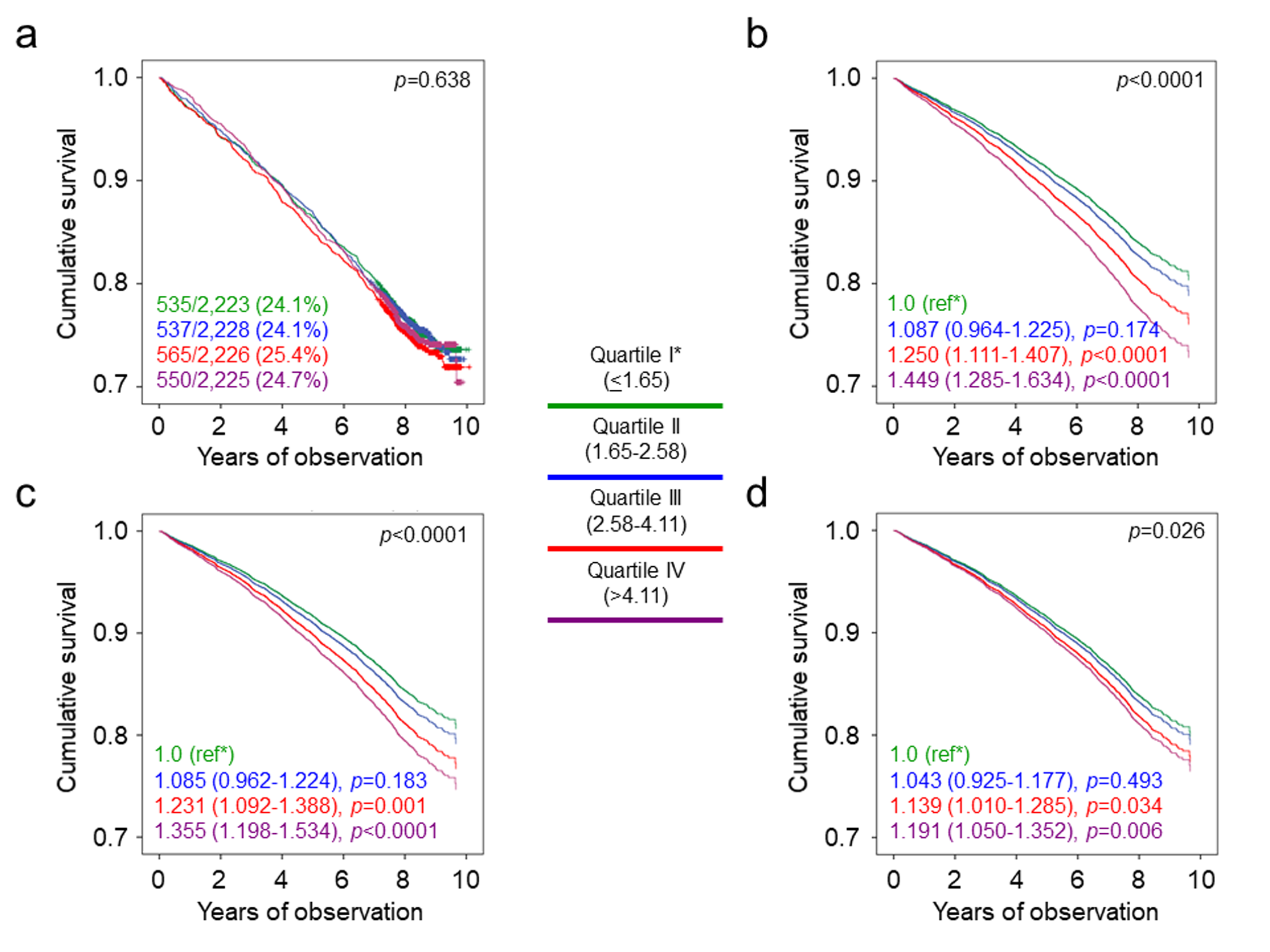
**

**Figure S5.** Survival analysis by quartiles of TG:HDL in males. Cumulative survival by Kaplan Meier-analysis (a) and Cox proportional hazards regression, adjusted for age and gender (b), plus CVD risk factors (c) plus complications/comorbidities (d), according to quartiles of TG:HDL ratio in males. Numbers (percentages) of deaths and HRs (95% CI) for mortality are shown for each group. TG:HDL = triglycerides:HDL cholesterol ratio; HR = hazard ratio; CI = confidence interval.

**
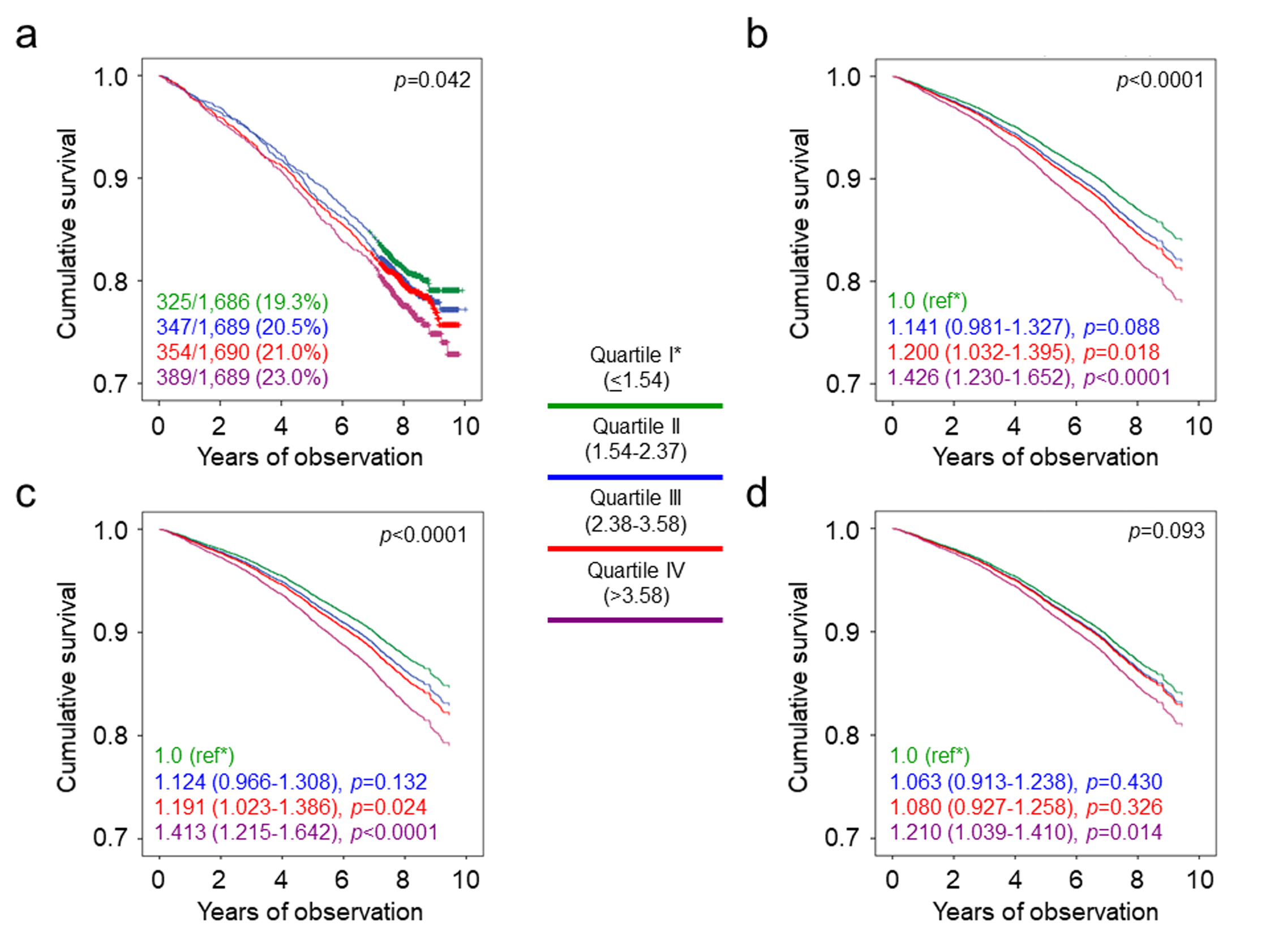
**

**Figure S6.** Survival analysis by quartiles of TG:HDL in females. Cumulative survival by Kaplan Meier-analysis (a) and Cox proportional hazards regression, adjusted for age and gender (b), plus CVD risk factors (c) plus complications/comorbidities (d), according to quartiles of TG:HDL ratio in females. Numbers (percentages) of deaths and HRs (95% CI) for mortality are shown for each group. TG:HDL = triglycerides:HDL cholesterol ratio; HR = hazard ratio; CI = confidence interval.
