# Supplementary material for: Independent association of atherogenic dyslipidaemia with all‐cause mortality in individuals with type 2 diabetes and modifying effect of gender: a prospective cohort study
Source: Cardiovasc Diabetol. 2021 Jan 30;20:28. doi: 10.1186/s12933-021-01224-7 (PMC7847015; doi:10.1186/s12933-021-01224-7)
Supplement: Supplementary file 3 — Additional file 3. The RIACE Study Group [file 12933_2021_1224_MOESM3_ESM.doc]

**Additional file 3: The RIACE Study Group**

**The RIACE Steering Committee**

Giuseppe Pugliese (Coordinator), Giuseppe Penno (Secretary), Anna Solini, Enzo Bonora, Emanuela Orsi, Roberto Trevisan, Luigi Laviola, Antonio Nicolucci.

**Participating Diabetes Centres**

1. Azienda Ospedaliera Sant'Andrea, Roma (Coordinating Centre): Giuseppe Pugliese, Lucilla Bollanti, Elena Alessi, Martina Vitale, and Tiziana Cirrito.
2. Ospedale Le Molinette, Torino: Paolo Cavallo-Perin, Gabriella Gruden, and Bartolomeo Lorenzati.
3. Ospedale San Luigi Gonzaga, Orbassano: Franco Cavalot, Mariella Trovati, Leonardo Di Martino, and Fabio Mazzaglia.
4. Ospedale San Raffaele, Milano: Giampaolo Zerbini, Valentina Martina, Silvia Maestroni, and Valentina Capuano.
5. IRCCS “Cà Granda – Ospedale Maggiore Policlinico”, Milano: Emanuela Orsi, Eva Palmieri, Elena Lunati, Valeria Grancini, and Veronica Resi.
6. Ospedale San Paolo, Milano: Antonio Pontiroli, Annamaria Veronelli, and Barbara Zecchini.
7. Ospedale San Giuseppe, Milano: Maura Arosio, Laura Montefusco, Antonio Rossi, and Guido Adda.
8. ASST - Ospedale Papa Giovanni XXIII, Bergamo: Roberto Trevisan, Anna Corsi, and Mascia Albizzi.
9. Ospedale Maggiore, Verona: Enzo Bonora, and Giacomo Zoppini.
10. Policlinico Universitario, Padova: Angelo Avogaro, and Monica Vedovato.
11. Ospedale Cisanello, Azienda Ospedaliero-Universitaria Pisana, Pisa: Giuseppe Penno, Laura Pucci, Daniela Lucchesi, Eleonora Russo, and Monia Garofolo.
12. Ospedale Santa Chiara, Azienda Ospedaliero-Universitaria Pisana, Pisa: Anna Solini.
13. Ospedale Le Scotte, Siena: Francesco Dotta, Cecilia Fondelli, and Laura Nigi.
14. Policlinico Umberto I, Roma: Susanna Morano, Tiziana Filardi, Irene Turinese, and Marco Rossetti.
15. Ospedale S. Maria Goretti, Latina: Raffaella Buzzetti and Chiara Foffi.
16. Ospedali Riuniti, Foggia: Mauro Cignarelli, Olga Lamacchia, Sabina Pinnelli, and Lucia Monaco.
17. Policlinico Universitario, Bari: Francesco Giorgino, Luigi Laviola, and Annalisa Natalicchio.
18. Policlinico Mater Domini, Catanzaro: Giorgio Sesti and Francesco Andreozzi.
19. Policlinico Monserrato, Cagliari: Marco Giorgio Baroni, Giuseppina Frau, and Alessandra Boi.
